# Deep learning based semantic segmentation of leukemia effected white blood cell

**DOI:** 10.1371/journal.pone.0320596

**Published:** 2025-05-08

**Authors:** Zahoor Jan, Muhammad Shabir, Haleem Farman, Afzal Rahman, Moustafa M. Nasralla

**Affiliations:** 1 Department of Computer Science, Islamia College University, Peshawar, Pakistan; 2 Smart Systems Engineering Laboratory, Department of Communications and Networks Engineering, College of Engineering, Prince Sultan University, Riyadh, Saudi Arabia; 3 Department of Mathematics, University of Peshawar, Peshawar, Pakistan; Xidian University, CHINA

## Abstract

Medical image segmentation has numerous applications in diagnosing different diseases. Various types of diseases are found in white blood and Red blood cells. This paper represents the segmentation of WBCs from blood smear images. It is a complex and challenging task due to the frequent overlapping and variants in size and shape of WBCs with each other and RBCs. This overlapping is due to the rough border of the immature cells. The paper describes a new approach to WBC segmentation using UNet++, the marker watershed algorithm, and Neural Ordinary Differential Equations (ODE). This technique uses UNet++ for pre-segmentation, followed by the marker watershed method, which has been integrated using ODE to deepen the segmentation process. This novel integration enhances clinical applications in automated blood cell analysis, diagnostic imaging, and disease monitoring, improving accuracy and robustness. The ODE is used after the convolution operation to reduce the error at each step, preventing the massive propagation of error in the forward and the backpropagation. The White blood cells are segmented from the input smear images using ALL_IDB1 and ALL_IDB2 datasets, which are further used in the experiment section. UNet ++ is used to generate the pre-segmented probabilistic grayscale images. Some white blood cells are connected and make groups appearing in the grayscale images. These groups of WBCs are separated using a technique called the marker watershed, which gives us the final segmented result. The experimentation results show that the mean intersection over union (Jaccard method), the Dice similarity coefficient, and the mean pixel accuracy are 97.73%, 98.36%, and 98.97%, respectively. The structure and size of the white blood cells vary from red blood cells and platelets, which makes this work different from others. Furthermore, the combination of UNet++, marker watershed, and Neural Ordinary Differential Equation makes the proposed system unique from existing systems. This work can be further investigated to reduce computational complexity and memory space for optimizing deployment on low-resource devices, such as smart healthcare systems. Techniques like model pruning, quantization, or learned information distillation might be explored to create a lightweight version of the model without much loss in accuracy. Such developments would make possible mass uses of automated white blood cell segmentation in portable, low-cost health devices for point-of-care remote diagnostics and monitoring.

## 1. Introduction

Leukemia is a kind of cancer of the blood and bone marrow with subsequent medical implications when normal production of blood cells is compromised, resulting in impaired immunity, increased infections, anemia, and the possibility of internal bleeding. Prompt diagnosis is always a key component to successful therapy; however, methods of examination, such as blood smear analysis and biopsy of the bone marrow, are often quite time-consuming and labor-intensive as well as with possibilities of errors in human calculations. Other techniques also use experienced technologists that can interfere and are not convenient for screening purposes or surveillance procedures. Consequently, there exists a demand at present for techniques that are quick, accurate, and more accessible than those currently applied for early diagnostics of leukemia cases across the globe.

Medical image processing research for leukemia treatment is crucial because earlier diagnosis and better monitoring of all the disease stages are possible. Given that image processing can be employed to recognize leukemia cells and categorize them, diagnosis can be made much more efficient and available in regions without access to many specialists. Image processing also helps monitor disease stages and modify the treatment as soon as possible, which is essential in chronic cases. It may also help to decrease the number of invasive solutions, which can be done by providing non-invasive solutions to diagnose blood and bone marrow samples. In general, the advancement of medical image processing in leukemia benefits from advanced diagnostics, better treatment, and, of course, patients’ life expectancy.

**Fig 1:**
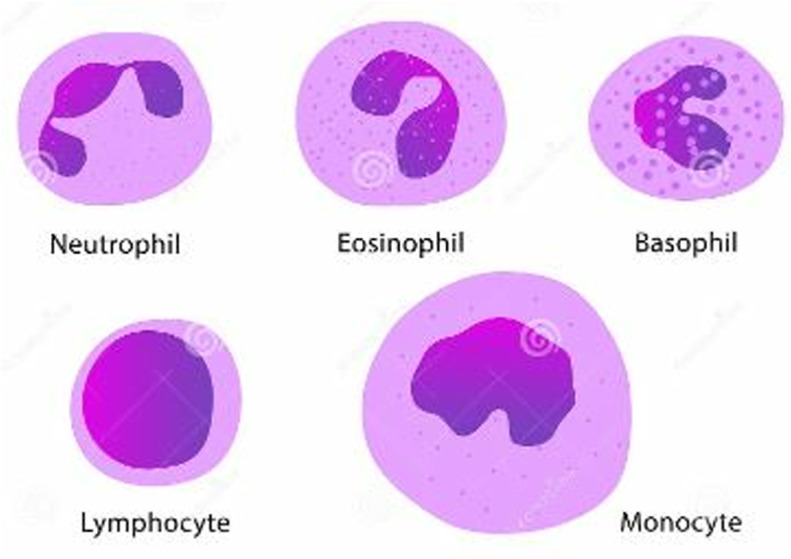
Types of White Blood Cell.

Human blood includes a variety of white cell types, as shown in (**Fig 1**), which produces several diseases, including Leukemia. Leukemia is a type of blood cancer that arises due to the rapid increase of immature WBCs. The immature white blood cells kill other cells, causing diseases.

The severity of the disease depends on several factors, including the patient’s age and health, genetics, the kind of Leukemia (chronic or acute), and the time of diagnosis. Acute Leukemias like ALL and AML, for example, can quickly develop into life-threatening diseases in a matter of weeks if left untreated. Chronic Leukemias, CLL, and CML, on the other hand, have a more subtle onset, and symptoms appear years later. While older individuals or those with additional conditions may be at greater risk, younger people have the most significant prognosis. Genetic alterations also play a role in the disease’s stepwise progression; some, like the Philadelphia chromosome mutation linked to CML, respond to focused treatment, whereas CLL and CML, being chronic leukemias, are more insidious in basing, and patients develop symptoms after several years. Younger patients have the best prognosis, whereas older patients (age-wise) or patients with other illnesses may have more risks. Stepwise progression of the disease also involves genetic mutations; some respond to targeted treatment, like the Philadelphia chromosomal mutation found in CML, whereas other mutations may predispose patients to a poor outcome.

However, in every case, the body’s ability to produce blood cells is impaired due to a propensity to Leukemia, eventually compromising the person’s health and quality of life by causing weakness, infections, and bleeding issues.

In the late 1990s, leukemia was diagnosed using a time-consuming and laborious laboratory test, and then automated techniques were developed [1]. The details of existing manual and clinical methods for diagnosing malignant WBSs are given in the following.

- Patient Medical History
- Cytogenetic Analysis
- Blood Cell Counting
- Manual Microscopic blood smear image analysis
- Immunohistochemestry of Blood Sample
- Biopsy
- Catheter Drainage
- Percutaneous Aspiration
- Long Distance Inverse Polymerase Chain Reaction
- Molecular Cytogenetics

Leukemia malignancy is diagnosed using various techniques, including patient medical records that help to reveal risk factors and symptoms. Cytogenetic analysis has been used to detect the presence of chromosomal abnormalities in leukemia. A blood cell counter gives a preliminary count of WBCs, while a cell differential count using manual microscopic blood smear analysis is a visual check of blood samples under a microscope. Immunohistochemistry is employed to detect specific markers on blood cells, and biopsy gives the chance to take bone marrow or tissue samples for direct examination. Consequently, other non-invasive techniques, such as catheter drainage and percutaneous aspiration, are also used to get samples for analysis. Long-range PCR (LRCR-PCR) can be a method of specific genetic mutation detection related to leukemia, and molecular cytogenetics can be a tool that studies those alterations at the molecular level. In contrast to the automated methods, the manual and clinical ones still significantly provide extensive insight into the disease.

**Fig 2:**
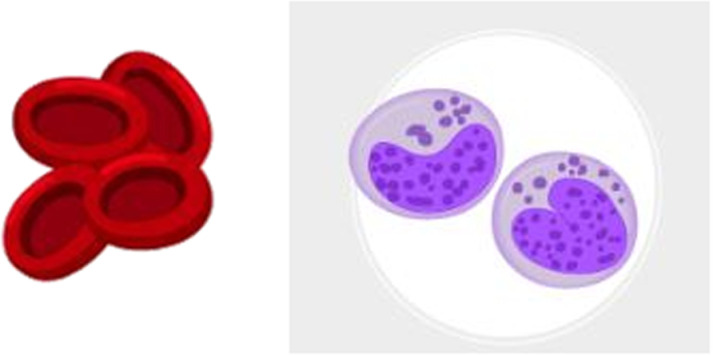
Normal Red and White Blood Cell.

The white blood cell ratio in the human body is 1000:1. The ratio indicates 1000 red blood cells for one white blood cell [2]. Therefore, leukemia illnesses will develop if the white blood cell count rises clearly and severely. If immature white blood cells increase, they will start destroying other cells. The normal red and white blood cells are shown in (**Fig 2**).

Various leukemia subtypes can result in multiple issues. Early on, you might not see any symptoms. The condition cannot be diagnosed solely by its signs and symptoms, but it can serve as indicators for you and your doctor to identify the issue as soon as feasible. If you experience symptoms, these could include Poor blood clotting, Red spots on the skin, Frequent infections, and Anemia. Leukemia symptoms include shortness of breath, night sweats, weakness or exhaustion, fever or chills, discomfort in your bones or joints, weight loss, and swollen lymph nodes or organs like your spleen. Leukemia can also manifest as symptoms in organs where the cancer cells have already invaded the tissue. For instance, headaches, nausea, vomiting, confusion, and seizures may result from cancer spreading to the central nervous system. In addition, leukemia can spread to your lungs, digestive system, heart, and kidneys, among other body organs.

The main objective is to segment all White blood cells, which can be further used to identify immature cells. It can also be useful in any emergency, and there might be a technique that quickly segments the white blood cells for different diseases. The proposed method can also be integrated with different smart health Apps in pandemic situations [3]. The suggested approach distinguishes between other blood cell types as well. Furthermore, it is crucial to do an analytical study on the ability to differentiate between various cell types, classify them as undeveloped cells, and then utilize that information to identify leukemia. The immature blasts of lymphoid and myeloid cells result in Lymphocytic and Myeloblastic conditions, respectively. Medical image analysis is now a trend to efficiently diagnose different types of diseases from blood in nearly real-time, either using optimized machine learning or deep learning techniques [4,5].

Segmentation is the division of an image into different sections based on certain shared characteristics. In medical imaging, segmentation helps examine anatomical structures, diagnose patients, formulate treatment plans, and identify malignancies and other conditions [6].

This work presents a novel deep-learning method for pre-segmenting cells from the blood through the integration of UNet++ [7]. A marker-based watershed (MW) approach is used to improve the segmentation of WBCs. This research study’s primary contributions are:

- A novel segmentation technique, which is the combination of U-Net++, ResNet, Neural Ordinary Differential Equation, and Marker Watershed.
- The MW algorithm produces the concluding segmented image of WBCs using the probabilistic grayscale map.

## 2. Literature

Many segmentation techniques have been reported for the identification and segmentation of blood cells and vessels [8–11]. To segment peripheral blood smears, Using the (KTE) Teager Energy operator is advised by Kumar et al. [12]. Here, the white blood cell (WBC) cytoplasm is separated using a selective mathematical operator, and a cell’s nucleus is segmented using edges, which (KTE) operator efficiently recognizes.

Piuri and Scotti examine the effectiveness of edge determination and contrast stretching for the automated identification of leucocytes [13]. Coupling contrast stretching, a clever edge detector, and a selected morphological operator allow for the detection of leucocyte membranes and nuclei. Issues related to image collection criteria and smear discoloration throughout the process of creating blood smears could potentially hinder the effectiveness of any previously stated segmentations. In aberrant blood cells, the round geometry hypothesis for cell segmentation is inappropriate [14].

Researchers have recently created two categories of different blood cell segmentation approaches. The majority of current algorithms segment data using both conventional and deep learning-based methodologies. These methods are intended to improve the handling of overlapping and clustered cells. Graph theory [15,16], level sets [17,18], the watershed method [19,20], and the region-growing approach are examples of traditional segmentation techniques. Convolutional neural networks, U-Net, U-Net++, and fully convolutional networks are the main types of deep learning methods. Significant for irregularly shaped and sized cells. An approach for segmenting clustered cells in classical cell images developed by Wang et al. [21] combines mathematical, morphology, and graph theory. The algorithm is simple, but the segmentation impact could be more optimal for irregularly shaped and sized cells, and the mistake rate is high.

To distinguish overlapping cells, the authors of the research [22] combined the region-expanding technique with color space transformation. This method’s mean pixel accuracy is excellent. However, the accuracy decreases when there are no visible internal borders among the clustered cells. Miao et al. [23] described a marker-controlled watershed technique to segment RBCs; we apply this approach to WBCs instead. After removing the binary images of the cells, foreground and background markers were first acquired. The watershed approach was then applied to the regenerated gradient topographic map to obtain segmentation results. Although the segmentation technique is simple to use and inexpensive to compute, its accuracy may be impacted by low-quality images.

Due to noise, performance, and other factors, certain traditional segmentation approaches cannot achieve high segmentation robustness. Compared to conventional image segmentation, deep learning methods have a higher segmentation accuracy because they use a large number of samples to learn the characteristics of cell images. Consequently, deep learning algorithms can be used in conjunction with the traditional segmentation technique to achieve semantic segmentation [24–27]. A method for segmenting partially overlapping cells that coupled U-Net was developed by Huang et al. [27]. This method extracted the cell contour using an improved level set energy function and employed U-Net to identify the cytoplasm and nucleus. This function increased segmentation accuracy by first incorporating a shape and a distance map. Kowal et al. [28] proposed integrating CNN with a seeded watershed approach to segment cell nuclei. This method uses a CNN classifier to pre-segment the cell nuclei. A seeded watershed is used to manage overlapping nuclei. Despite the high computational complexity, their results outperform conventional watershed approaches.

In the paper [29] the authors segmentized images with the presence of noise. They used power mean in their approach to segmentize different types of images, including medical images. They claimed that the power mean function is robust in the presence of an outlier. They also used the fuzzy membership function and power means to strengthen the segmented process further. Another recent research work has been done by Makem et al. [30]. They segmentize the nuclei of WBC and can be further used for different types of diseases. Their proposed system uses the mean method to smooth the input image. They enhanced the texture of images using arithmetic operations with the fusion of Fourier transformation. The nuclei of WBCs are extracted using the k-mean technique. They claimed 97.35% accuracy on CellaVision and 93.48% ALL-IDB2 database. Resendiz et al. [31] use U-Net for WBC segmentation and ResNet50 for classification. Their system achieves a segmentation accuracy of 91% and a classification accuracy of 99.9%. Wei Liu [32] et al. investigate deep and traditional Machen learning models for bacterial pathogens diagnoses. They claimed that deep learning techniques, i.e., (CNN) are better than conventional machine learning techniques in bacteria detection. After detailed experimentation, they justify their claim by obtaining accuracies. According to their proposed architected, 99.6%, 98.9%, and 96.4% accuracy are achieved by CNN, SVM, and Random Forest, respectively.

Shoaib et al. [33] focus on automated segmenting of the left ventricle (LV) from 2D echocardiographic images, an essential task in medical imaging for diagnosing cardiovascular diseases. The performance of three advanced architectures of CNN, Fully Convolutional Network (FCN), SegNet, and Mask R-CNN for segmenting the LV are evaluated in this study. A key finding of this paper is the development of echocardiography images. In addition, the performance of each model in terms of segmentation is evaluated using several evaluation metrics: pixel accuracy, precision, recall, specificity, Jaccard index, and Dice similarity coefficient (DSC). They claimed that Mask R-CNN consistently outperformed SegNet and FCN across all metrics among the three models. The Mask R-CNN model showed a high DSC of 92.21%, a Jaccard index of 85.55%, and excellent values for other measures such as precision (93.15%) and recall (96.81%) while being trained with 4,000 images. However, the model’s performance becomes stable when the training data exceeds 4,000 images. The paper [34] explores the application of deep learning in interpreting SPECT scans, specifically for Parkinson’s Disease (PD), using DaTscan imaging. This research demonstrates the ability of deep understanding, transfer learning, and soft attention mapping to differentiate between normal versus PD cohorts with DaTscan SPECT images. The approach is not feasible for analyzing the motor outcomes of such scans. The paper employs DenseNet-121 as the main model with a soft attention block, achieving 99% AUC and 99.2% accuracy. This is greater than other CNN models, such as Xception and ResNet. The paper uses soft attention mapping rather than Grad-CAM because of its cost-effectiveness and the ability to point out the relevant features, even with a small sample size. The approach is semi-automatic and not fully automated; therefore, an even more significant 3D CNN could better capture features of the whole scan volume. Future studies should develop an end-to-end 3D CNN model for more effective feature extraction from 3D SPECT data while increasing the number of samples in the database for robust outcomes. The research paper [35] introduces a new federated epistasis detection framework, FedED-SegNAS, to handle the challenges of detecting disease-associated genetic variants in a privacy-preserving manner with improved communication efficiency in multi-institutional genome data sharing. This framework uses FL to allow several institutions to collectively analyze genomic data without having to share the data directly. Sequence perturbation privacy-preserving is used to ensure privacy. It protects the data during the parameter transfer by grouping and randomly perturbing parameters, making it hard for attackers to link parameters to specific groups. Fuzzy logic is incorporated into convolutional neural networks (CNNs) to improve the interpretability of deep learning models. The model can deal with the ambiguity of genomic data, maintaining high interpretability and accuracy. The framework uses a NAS method based on PSO to automatically search for the optimal neural network architecture at different stages of federated learning. This optimization reduces the communication cost between institutions and improves communication efficiency.

## 3. Proposed methodology

**Fig 3:**
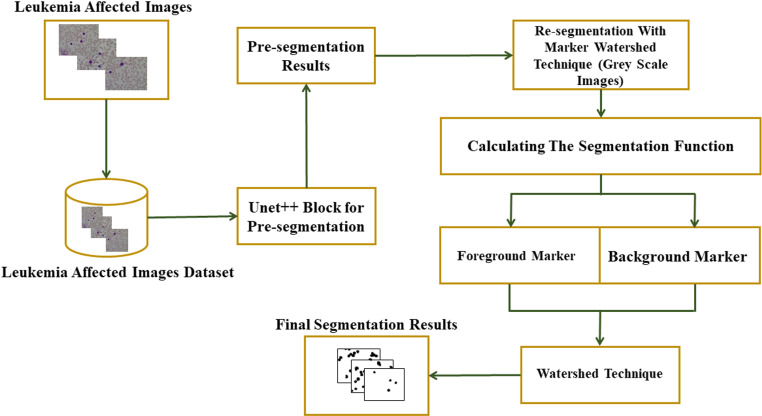
The Proposed Framework.

This section introduces UNet++ and the marker watershed-based approach to segmenting WBC images. (**Fig 1**) depicts the proposed framework conceptual structure. The WBC images are segmented using the deep learning network UNet++, and the model is trained using the labeled training set. The image is segmented using the learned model to create the probability grayscale image. The final segmentation findings of WBSs are achieved after further optimizing the pre-segmentation results using an MW method, as given in (**Fig 3).**

The proposed system uses ODE for error reduction. An ODE is a differential equation involving the derivative of one or more dependent variables with respect to a single independent variable.


$$\frac{dg(k)}{dk}=f(g(k),k)$$ (1)

In equation (1), k is an independent variable, g(k) is an unknown function (dependent variable), and the derivative of the anonymous function is referred to as an ordinary differential equation (ODE). The left-hand side of equation (1) is the derivative of g with respect to k while the right-hand side of equation (1), f(g(k),k) is a function of g and k. It is more typical in engineering to solve the equation numerically. One simply needs to approach the unknown value gradually with a known value rather than trying to solve the general solution of the anonymous function. Euler’s method is a common technique for resolving the numerical value of differential equations, which is given in equation (2).


g(k + Δk) = g(k) + Δk × f(g(k), k)  (2)

In equation (2) Δk is a small step in k. The small change or step Δk causes changes in g. Furthermore, it reduces the error in each convolution. As the process progresses, errors are produced at every step, mounted to solve differential equations, and the ODE solver is used. It travels with a defined step size as opposed to the Euler technique. It chooses an acceptable step size based on an established error tolerance to approach an unknowable value.

**Fig 4:**
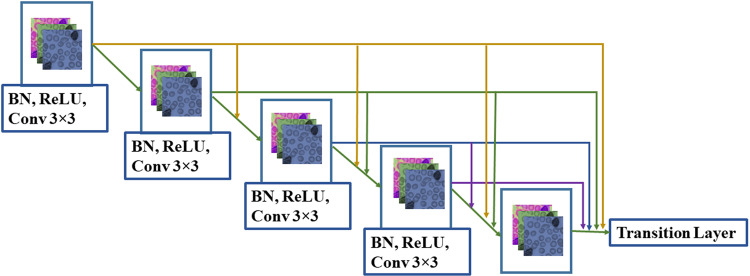
Generic structure of ResNet.

A deep learning technique called residual network (ResNet) is used in the proposed framework. The ResNet has two types of connection, i.e., residual and direct connection. The generic structure of ResNet having a dense layer is given in (Fig 4).

**Fig 5:**
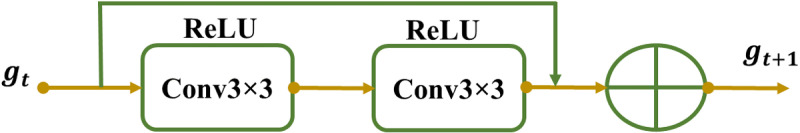
Residual block structure.


Fig 5 shows the residual block architecture, and a residual network (ResNet) [36] is a specific type of NODE.

$$g_{t+1}\;=\;g_{t}+\;ℱ(g_{t},\;\rho_{t})$$ (3)

**Fig 6:**
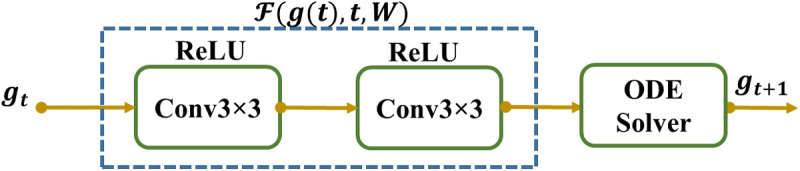
ODE block Structure.


Here, the resultant vector $g_{t}$ is from the layer t, the network parameters $\rho_{t}$ of the layer t, and ℱ is the layer t parameterized neural network (NN). In (3), ℱ in ResNet comprises convolutional processes. The ResNet can be observed as an Euler discretization if t = 1. As Δt → 0 diminutions, a steady convergence with more layers added in smaller increments is the result. This limit state transforms into an ODE designated by the neural network, as shown in (Fig 6).

$$\frac{dg(t)}{dt}=\lim_{\Delta t\to 0}\frac{g_{t+\Delta t}-g_{t}}{\Delta t}=F\left(g(t),t,\rho \right)$$ (4)

An ODE solver may solve a continuous neural network in which the forward pass operations are very straightforward, and the ODE solver backpropagation, whose precise function can be found in [7].

### B. UNET++

**Fig 7:**
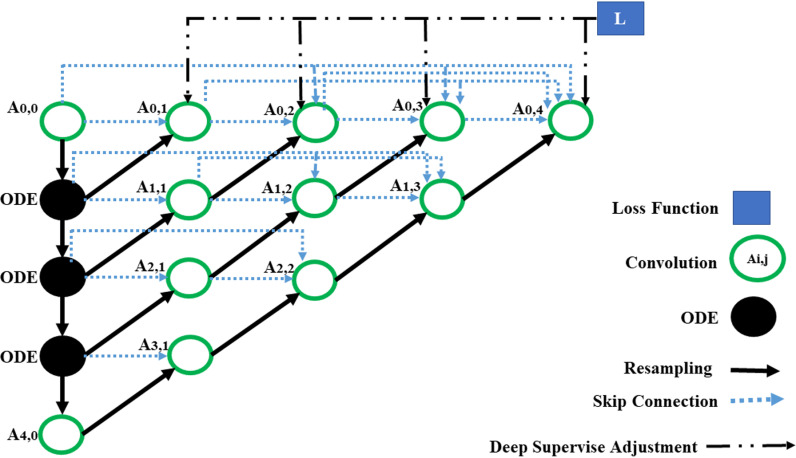
Architecture of the proposed technique.


In a UNet++ architecture, the suggested pre-segmentation model takes advantage of the ODE solver, as shown in (Fig 7), which displays the model in detail. There are two primary ways to differentiate the proposed technique from traditional U-Net++. First, the shallow feature maps are extracted using convolutional and ODE blocks in the encoder stage. Another difference is the addition of several convolutional layers by the U-Net++ and momentary connections between the encoder and decoder layers. Long links are utilized throughout the U-Net feature extraction process to combine surface features calculated by the encoder, while the decoder module extracts deep features. U-Net++ uses skip connections in the proposed technique to lessen the semantic difference between deep and surface features. It is challenging for U-Net to combine superficial feature images and deep feature images. Using the skip connections technique in dense convolutional blocks of UNet++, which U-Net++ influenced, fills in the semantic gap between the encoder and decoder feature maps. Like traditional U-Net++, the proposed technique employs deep supervision and augments each output node with a 1×1 convolutional layer and a sigmoid function. The sigmoid function transfers the output value u to [0,1], with a value range of [0,1], as indicated in equation (5).

$$\varphi (u)=\frac{1}{1+e^{-u}}$$ (5)

The degree of discrepancy between the model’s true values and predictions is measured using the loss function. The more closely the predicted value of the model matches the actual value, and the more robust the model is, the smaller the loss value of the function is during the neural network training process. The challenge of white blood cell segmentation is binary. As a result, we use binary cross-entropy loss, which is given below.


$$loss\;=\;-\frac{1}{M}\;\sum_{i=1}^{M}[\xi_{i}\;log\;p(\xi )+(1-p(\xi ))\;log\;(1-p(\xi_{i}))\;]$$ (6)

In (6) M stands for the total number of pixels in the image, ξ for the training label, which indicates whether pixel ξ is a white blood cell or not, and p(ξ) for the likelihood that ξ labels will be produced.

### C. MARKER WATERSHED ALGORITHM

Vincent et al. implemented the traditional watershed technique of segmenting regions of an image in 1991. Its fundamental concept is to think of the image as a topographic surface. The image’s pixel with the grey value of 0 depicts the ground, the pixel with the largest value, the highest point of the terrain, and the lines connecting the larger grey-valued pixels. White blood cell images contain more than one extreme pixel; hence, applying the watershed algorithm directly to these images frequently results in over-segmentation. The MW algorithm enhances the conventional watershed method by locating several backdrop and foreground indicators that segment cell images more accurately. Therefore, the WBCs are divided using the MW method. Initially, the WBC gradient images should be extracted and processed. After creating the foreground, adjust the gradient image. Markers, followed by background markers, divide WBCs before applying the watershed algorithm.

The gradient image serves as a segmentation function for the MW method, reflecting information about the grayscale variation on the pixels. The gradient of the white blood cell image is obtained using a Sobel operator [37], which performs lateral and vertical detection of the WBC using two sets of 33 matrices and a convolution function. Although the calculation is quick and easy, edge discontinuities necessitate applying morphological reconstruction to WBC gradient images. Reconstructing open and closed processes allows for the processing of WBC images. While morphological restoration can better preserve the morphology of WBCs, open and closed operations can soften the edges of WBCs. The proposed system produces a planar disc-shaped structural element with a radius of three.

The *imregionalmax* function is utilized to locate the local maximum for the image to retrieve the foreground marker. A WBC image is transformed into a binary image after morphological processing, and the watershed is carried out after the distance transformation of the binary images. The background reference is the watershed ridge. The local minimum only exists at the marker places when the WBC gradient picture is corrected using the imimposemin function. The background marker is spread on the image background, and the foreground marker is assigned to a cell, providing a one-to-one relationship that successfully suppresses the over-segmentation problem. The marker watershed algorithm is then applied to the rectified images for the final WBC segmentation outcomes.

## 4. Experimental results

### A. Results

**Fig8:**
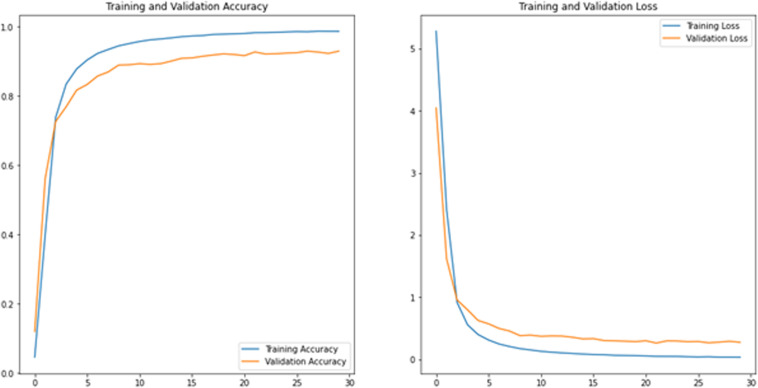
(a) The accuracy of the training and validation set (b) The losses in the training and validation set.

The effectiveness and robustness of the recommended method are empirically assessed and proven using a large number of 256 × 256-pixel images. All of the Images can be found in the Leukemia dataset, i.e., ALL_IDB1 and ALL_IDB2. A total of 100 images were picked from the datasets and divided into two sections, i.e., training and validation sets, with a ratio of 80% and 20%, respectively. The primary research work is the semantic segmentation of white blood cells, but classification and validation are also performed to check whether the segmentation is useful. The proposed method comprises a deep learning architecture called ResNet, in which Adam optimizer is used for optimization, and the learning rate is set to 1e-4. (Fig 8) exhibits the training loss, training accuracy, validation loss, and validation accuracy for each epoch during the training process. The proposed model is executed up to 30 epochs on the training and validation set. (Fig 8) (a) shows the accuracy of training and validation, which is quite remarkable. (Fig 8) (b) shows training and validation losses up to 30 epochs.

**Fig 9:**
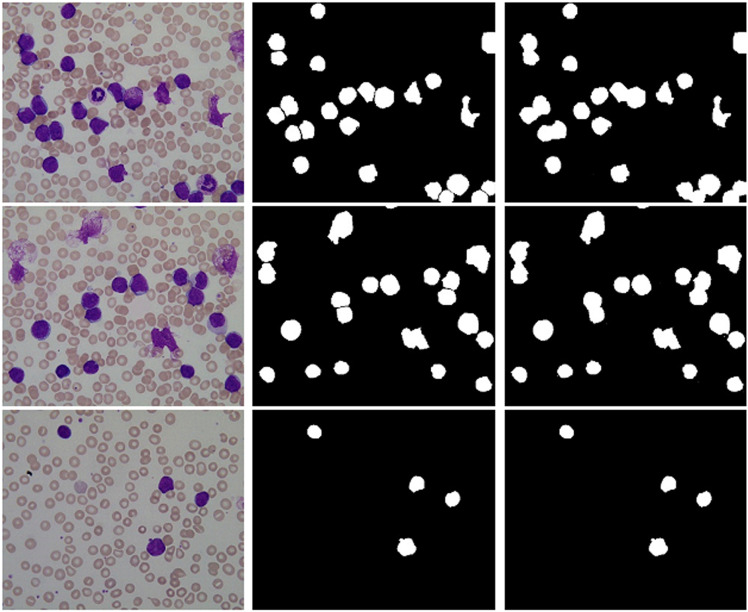
(a) Orignal Image (b) Label Images (c) Pre-segmentation using Unet++.

**Fig 10:**
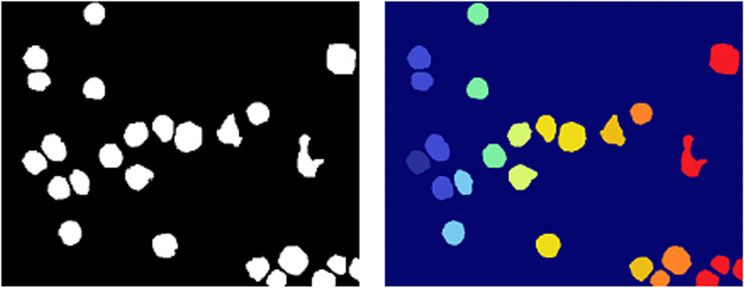
(a)^‌‌^ Foreground Marker (b) Segmented White Blood Cells.

Three examples of WBC pre-segmentation using the proposed technique are shown in (Fig 9). The original image is shown in (Fig 9a) the label image is shown in (Fig 9b) and the grayscale image obtained from pre-segmentation is shown in (Fig 9c). The first step segmentation result is further segmentized using the MW algorithm, as shown in (Fig 10) where (Fig 10a) displays the foreground marker image, and (Fig 10b) displays the segmented WBCs.

### B. Experimental analysis

**FIG 11:**
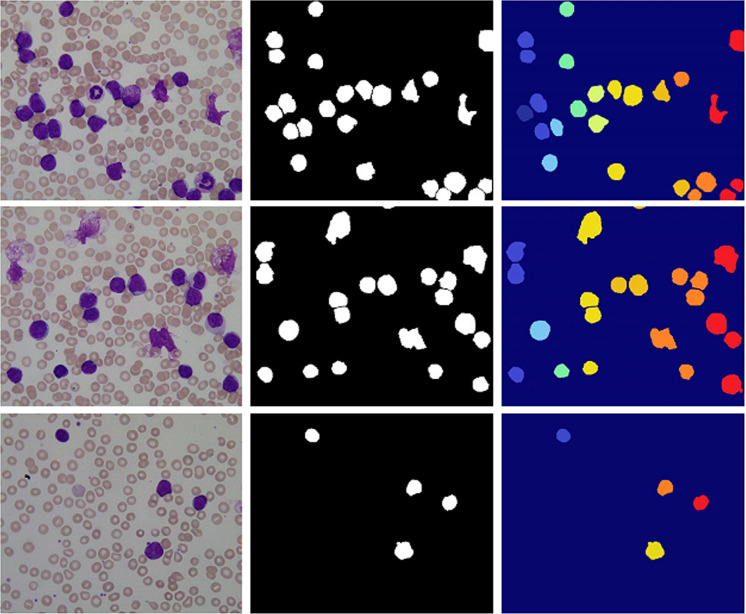
(a) Original ima ge (b) Label image (c) Final Result.

(Fig 11) shows the various conditions of blood smear images during segmentation. The original images from the dataset are shown in (Fig 11a) the label image is shown in (Fig 11b) and the segmentation image is showning (Fig 11e) using the fusion of UNet++, ODE, and MW algorithm.

(Fig 11) shows that the proposed method does an excellent job of extracting the contour of erythrocytes, but it significantly under-segments areas like clustering and overlapping WBCs. The MW algorithm may effectively tackle the erythrocytes problem, but it frequently results in missing extraction due to its sensitivity to weak edges and other interference factors. The Proposed technique can extract the majority of erythrocytes with various sizes, clusterings, and overlaps by combining the benefits of the two approaches, i.e., UNet ++ and MW. The erythrocytes hold a more consistent structure than WBCs, which display a more distinct appearance by most likely taking the form of tapered or irregular shapes. Additionally, there is a tendency for them to lump together as well instead of existing separately. The new approach that has been proposed in this paper is able to detect overlapping or clustered WBCs and regard them as a single object in the image. Though this technique is used specifically for WBC cell segmentation, with slight modifications, it can be used for red blood cell detection and diseases associated with red blood cells. The experimental findings are studied qualitatively and quantitatively to assess the proposed technique’s segmentation results further.

The segmentation accuracy of the proposed technique for WBCs is computed using various evaluation methods. This study has evaluated the segmentation outcomes of three methods using DSM, MPA, and MIoU. The DSM is a dice similarity measure, MPA is mean pixel accuracy, and MIoU is the mean intersection over union (MIoU). Measurements are based on the confusion matrix, as indicated in Table 1.

**Table 1:** Confusion matrix.

Sample	Positive	Negative
Actual Positive	**TP**	**FN**
Actual Negative	**FP**	**TN**

DSM is a predetermined similarity metric frequently used to compare two samples. Its value might be 0,1 or between 0 and 1. When the DSM value approaches 1, the performance increases. The DCM value near 1 indicates better segmentation outcomes. The Dice similarity measure (DSM) is given in (7).


$$\;DSM\;=\;\frac{2TP}{FP+FN+2TP}\;$$ (7)

Pixel accuracy (PA) is another technique used for performance measurement. It is the percentage of pixels correctly identified as erythrocytes and the number of pixels correctly identified as non-erythrocytes concerning the total number of pixels. In short, PA is the number of correctly predicted pixels as a percentage of the total number of pixels, as given in (8).


$$PA\;=\;\frac{TP+TN}{TP+FP+TN+FN}$$ (8)

The proposed system is also evaluated using mean pixel accuracy (MPA) (9). Mean pixel accuracy across all categories relates to the average pixel accuracy for white and non-red cells.


$$MPA\;=\frac{\frac{TP}{TP+FP}+\frac{TN}{TN+FN}}{2}$$ (9)

One recurrent metric used in semantic segmentation is intersection over union (IU). It refers to the intersection and union ratio between a class of models’ true and predicted values, which is given in (10).


$$IU\;=\;\frac{TP}{TP+FP+FN}\;$$ (10)

The average of the IU of erythrocytes and nonerythrocytes given in (11) is used as the mean value for each category of MIU.


$$MIU\;=\frac{\frac{TP}{TP+FP+FN}+\frac{TN}{TN+FN+FP}}{2}$$ (11)

**Fig 12:**
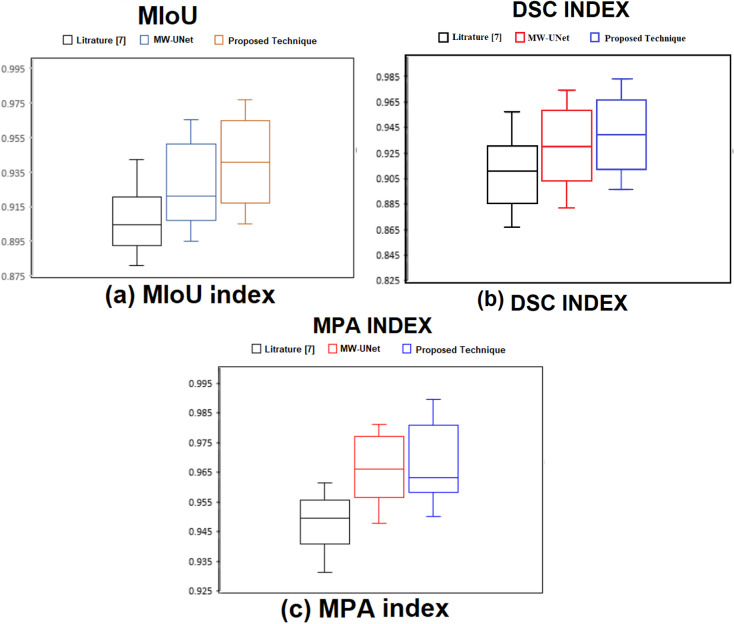
The box-plots of three measure segmentation accuracy.

Table 2 demonstrates the quantitative and qualitative findings from the same dataset, and (Fig 12) is the graphical representation (box plots) for the MIoU, DSC, and MPA. The outcomes demonstrate that this method for WBC segmentation is more efficient than the other available techniques.

**Table 2:** Quantitative results of three algorithms.

Model	MIoU	DSC	MPA
Technique in [7]	94.23%	95.77%	96.15%
MW-UNet algorithm	96.54%	97.43%	98.13%
Proposed Algorithm	97.73%	98.36%	98.97%

Several practical approaches can be considered to ensure the successful integration of the semantic segmentation system for white blood cell (WBC) smear images with existing hospital databases, digital pathology platforms, and automated microscopy equipment. These integration methods should aim to minimize disruption to current workflows and optimize clinical processes. Below are proposed ways to achieve this integration:

### C. Integration with hospital databases (LIMS)

The proposed semantic segmentation technique can be connected to the hospital’s Laboratory Information Management System (LIMS) to streamline data flow and ensure seamless patient and image d management. The possible practical integration methods are API and File format compatibility. The API can connect the proposed segmentation system with LIMS. This would allow the system to communicate with the database and automatically populate patient records with the results of WBC segmentation (i.e., segmented images and calculated metrics). In File format compatibility, The system can be designed to export segmented images in widely accepted formats (such as DICOM, TIFF, or PNG), ensuring compatibility with existing digital pathology platforms and hospital databases. Impact: By automating data entry, healthcare professionals can access the segmented images and associated diagnostic information directly within the patient’s record. This minimizes manual data entry and improves the speed and accuracy of accessing results.

### D. Integration with digital pathology platforms

The segmentation system should be designed to integrate directly with digital pathology platforms widely used in clinical labs for storing and reviewing medical images. This could include platforms like Aperio, Leica, or Philips, which allow pathologists to analyze high-resolution digital slides. Plugin or Extension and Cloud-based Solutions are possible ways to integrate with Digital Pathology Platforms. The proposed segmentation technique can be added as a plugin or extension to the existing digital pathology platform using the plugin or extension integration method. This would allow pathologists to run the WBC segmentation directly within the platform interface without switching between different systems. If any platform supports cloud-based solutions, the proposed segmentation technique can process the images and upload the results to the cloud, automatically synchronizing with the digital pathology platform. This would enable pathologists to access the segmented images remotely, further enhancing collaboration and flexibility in diagnostics. Impact: The integration with digital pathology platforms will save time by automating the segmentation step, allowing pathologists to focus on the diagnostic interpretation of images rather than manual segmentation. It will also provide a consistent and reproducible method for WBC identification, ensuring standardized results across different clinical settings.

### E. Integration with automated microscopy equipment

Automated microscopy equipment, such as slide scanners and digital microscopes, captures high-quality images of blood smear slides. These systems often have their own image-processing capabilities but can be enhanced by integrating the proposed WBC segmentation system. The Proposed semantic segmentation technique can be employed as a pre-processing step after the automated microscope captures the image. The system would automatically process the image, segment the WBCs, and generate the probabilistic or grayscale output, which is then passed on to the pathologist or diagnostic software for further analysis. The segmentation system could be integrated directly into the microscope’s software in a more advanced setup. After the microscope captures the image, the system processes the image in real-time, providing pathologists with an immediately segmented result that can be reviewed and analyzed further.

### F. Minimizing disruption to existing clinical processes

The integration process should prioritize ease of use. The segmentation system can have a simple user interface (UI) that complements existing platforms. For example, a button could be added to the digital pathology platform’s interface that triggers the segmentation process, displaying the results alongside the original images. The system should be intuitive and require minimal additional training for healthcare professionals. By ensuring that the segmentation output is automatically integrated into the platform and presented in a familiar format, the transition will be smooth, and clinicians can continue using the tools they are accustomed to. The segmentation results should be synchronized with patient records and accessible across different systems (e.g., LIMS, digital pathology platforms, and microscopes). This can be done through cloud storage or automatic synchronizing protocols that guarantee clinicians access to the latest information on every platform they use.

### G. Making the clinical workflow more efficient

The system automatically segments the WBCs, significantly reducing clinical officers’ time segmenting and analyzing images manually. It thus translates into quicker diagnoses, better turnaround times and perhaps more rapid decision-making in case of emergencies. Adopting the proposed semantic segmentation technique will help maintain diagnostic accuracy by minimizing human intervention error and providing a dependable, reproducible approach to WBC segmentation. This is particularly important in improving diagnostic accuracy, particularly when assessing the morphology of cells that appear to differ slightly. The idea is that the movement of the advanced technique to cloud-based platforms allows the collaboration of many healthcare staff, including pathologists, clinicians, and laboratory specialists, whereby segmented images and diagnostic views are sent to each other from different places.

## 5. Conclusion

This work proposes semantic segmentation of white blood cells based on UNet++, a DensNet-based technique, and the MW algorithm to address the issues of WBC clustering and overlapping With the integration of ODE (Orderly Differential Equation). The DensNet-based UNet++ network is specifically built to extract image features using an encoder concept. The skip connections in the proposed technique close the semantic gap between surface and deep feature maps. The features from the encoder are concatenated with the input from the corresponding skip connection in the decoder part. The ODE is embedded after convolution to calculate small values to reduce errors and prevent the model from propagating big values as errors. The experimental results demonstrate that compared to the MW method and the UNet++ algorithm separately, the UNet++ and WM, in combination with the integration of ODE, are efficient in segmentizing WBC. The proposed technique achieves segmentation accuracy of 98.36 on DSM, 98.97 on MPA, and 97.73 on MIU, which are quite high than the existing techniques given in the experimentation section. Furthermore, the proposed technique efficiently segmentized the immature, blasted, and overlapping WBCs. Even though the results of the proposed technique are encouraging, they can be enhanced using some other deep learning technique. The limitation of the proposed technique in its current state is that it is computationally complex. The next step is to make the system lightweight for use in low-resource devices, i.e., smartphones. The proposed technique can be used for decision-making in the digital laboratory. Soon, research will also be conducted to develop a technique suitable for Mobile Digital laboratories.
